# Effect of Job Strain on Job Burnout, Mental Fatigue and Chronic Diseases among Civil Servants in the Xinjiang Uygur Autonomous Region of China

**DOI:** 10.3390/ijerph14080872

**Published:** 2017-08-03

**Authors:** Suzhen Guan, Xiadiya Xiaerfuding, Li Ning, Yulong Lian, Yu Jiang, Jiwen Liu, Tzi Bun Ng

**Affiliations:** 1 Department of Social Medicine, College of Public Health, Xinjiang Medical University, Urumqi 830011, China; clever2066@sina.com (S.G.); xiadiye998@163.com (X.X.); 2 Department of Occupational Health and Environmental Health, College of Public Health, Ningxia Medical University, Yinchuan 750000, China; 3 Department of Occupational Health and Environmental Health, College of Public Health, Xinjiang Medical University, Urumqi 830011, China; nl96979@163.com (L.N.); yuyu_jiang88@126.com (Y.J.); 4 Department of Occupational Health and Environmental Health, College of Public Health, Nantong University, Nantong 226000, China; lianyulong444@163.com; 5 School of Biomedical Sciences, Faculty of Medicine, The Chinese University of Hong Kong, Hong Kong, China

**Keywords:** job strain, job burnout, mental fatigue, chronic diseases, civil servants

## Abstract

Job strain is a major concern in view of its effects among civil servants associated with job burnout, mental fatigue and chronic diseases. The objective of this study was to assess the job strain level among civil servants and examine the effect of job strain on job burnout, mental fatigue and the resulting chronic diseases. A cross-sectional study with a representative sample consisting of 5000 civil servants was conducted from March to August 2014. Using a structured questionnaire, the job strain level, job burnout and mental fatigue were measured by using the Personal Strain Questionnaire (PSQ), Maslach Burnout Inventory (MBI) and Multidimensional Fatigue Inventory (MFI-20), respectively. Overall, 33.8% of the civil servants were found to be afflicted with high and moderate job strain. The characteristics of most of the civil servants with a higher-job strain level were as follows: female, Uygur, lower educational level and job title rank, shorter working experience, married marital status, and lower income level. Civil servants suffering from chronic disease mainly had hypertension and coronary heart disease, which accounted for 18.5% of the diseases. Civil servants with a high-job strain level exhibited higher rates of burnout, mental fatigue scores and incidence of chronic diseases. There was a multiple linear regression model composed of three predictor variables in job burnout, which accounted for 45.0% of its occurrence: female gender, lower-income level, higher-job strain in civil servants, the greater the rate of job burnout was. Four factors—male gender, lower-job title rank, higher-job strain, shorter-job tenure of civil servants—explained 25.0% of the mental fatigue model. Binary logistic regression showed that intermediate-rank employees (OR = 0.442, 95% CI: 0.028–0.634; *p* < 0.05), job tenure of 10–20 years (OR = 0.632, 95% CI: 0.359–0.989; *p* < 0.05), and low-job strain (OR = 0.657, 95% CI: 0.052–0.698; *p* < 0.05) were all associated with significantly lower odds of chronic disease. The risk of chronic disease was higher in civil servants with high-job burnout scores and mental fatigue scores compared with civil servants with lower scores (OR = 1.139, 95% CI: 1.012–3.198; OR = 1.697, 95% CI: 1.097–2.962). These data provide evidence for the effects of job strain on job burnout, mental fatigue and chronic diseases among civil servants.

## 1. Introduction

Job strain refers to a state of physical and mental strain caused by an imbalance between strict job requirements and an inadequate ability to adapt and cope [1]. At present, different types of workers face a variety of job strains. An increased risk of job strain is associated with mental disorders, chronic diseases (such as hypertension [2], cardiovascular diseases [3], and diabetes [4]) and depression [5,6].

A number of workers suffer from mental disorders in China [7,8,9]. Civil servants are professionals with a high risk of mental disorders [10]. The term civil servants refers to personnel who perform their duties according to law, and are included in the national administrative system, whose salaries are paid by the state. They are responsible for managing state affairs and the liaison between citizens and the government [11,12]. According to the “Provisional regulations of the state civil servants of China”, civil servants are divided into those in administrative positions, administrative law enforcement, law enforcement, and professional and technical categories, such as judges, prosecutors, and police, etc. Extensive research has identified job-related stressors that contribute to job stress, including poor working conditions, ambiguous career roles, tight relationships, lack of autonomy, and higher work intensity [13,14,15]. Before the civil servant commence their jobs, they have to undergo a stringent occupational health examination, hence they are relatively healthy to start with, however, the nature of the work and the workplace environment determine the particularity of the work, which is different from other occupations, and civil servants encounter a variety of serious and sustained tensions in response to their specific performance [16]. In fact, a sizeable number of studies have disclosed that the general health condition of civil servants is not satisfactory, and can even be alarming [17], due to their job roles and types of job strain to which they are exposed [12].

Job burnout is a state of stress that is an important issue worldwide, which is characterized by emotional exhaustion, cynicism, and negative self-evaluation [18]. Over two decades of research on burnout have identified a plethora of organizational risk factors across many occupations in various countries [18,19]. When there is conflict of values on the job, and thus a gap between individual and organizational values, employees will find themselves making a trade-off between work they want to do and work they have to do, and this can ensue in greater burnout [20]. Job burnout is the sequel of chronic stress, typical of day-to-day work that has a direct impact on the efficiency and quality of work [21]. Burnout has a complex pattern of relationships with health, in that poor health contributes to burnout and burnout contributes to poor health [22]. As a consequence of job strain, it can also affect physical and mental health, and bring about mental exhaustion, physical fatigue and feelings of diminished competence [23]. Accordingly, the unhealthy psychological conditions of civil servants may result from their job burnout, and long-time job burnout can in turn lead to mental disorders. Mental fatigue is another important factor in the development of physical and mental illnesses, especially psychological diseases. The more serious the level of job strain, and the longer the duration of job strain, the more likely it will lead to psychological fatigue. In other words, the degree of psychological fatigue is positively correlated with the level of job strain.

There are accumulated voluminous data on the relationship between job strain and illnesses, such as, cardiovascular diseases, respiratory diseases, sleep disorders, coronary disease, cancer, hypertension, diabetes, stroke, and so on [24,25,26]. A cluster of several chronic diseases and higher scores on strain symptoms appeared on the reports of some employees. For this reason, and in order to explore the mechanisms for the association between job strain and health, we hypothesized that civil servants under greater job strain would have more intense job burnout and increased mental fatigue, and also suffer from a higher morbidity of chronic diseases.

An understanding of the factors associated with the level of job strain is necessary for explaining the health deterioration in civil servants and designing a strategy of interventions to better their health status. However, very few of such studies have focused on civil servants and studies have seldom quantitatively described the physical and mental health among civil servants in China. Therefore, the present study had two main research objectives: (i) to acquire data about the level of job strain among civil servants in the Xinjiang Uygur Autonomous Region of China; and (ii) to elucidate whether higher-job strain was an important factor influencing job burnout, mental fatigue and morbidity of chronic diseases among them. The end goal was to provide useful information for the development and implementation of health improvement strategies for this group of workers.

## 2. Methods

### 2.1. Subjects Studied

This cross-sectional study was carried out during the period between March and August 2014. In the study, a three stratified cluster sampling method was used to extract samples as follows. According to different geographical and economic conditions, the 14 prefectures and cities in Xinjiang were divided into four layers: prefecture-level cities; Eastern Xinjiang, Southern Xinjiang, and Northern Xinjiang. According to population proportion of different cities, we took institutions as sampling frames and randomly selected 5–8 institutions from each region, respectively. 100–200 civil servants of institutions were recruited by applying computer-generated random numbers to the city’s list of institutions. Finally, 5700 civil servants of the participants were well informed about the investigation and recruited as subjects and entered into the sample size. The survey was conducted in Urumqi (*n* = 1600) and Kelamayi (*n* = 1000) which are located in the north, He Tian (*n* = 800) and Aksu (*n* = 1000) located in the south and Tulufan (*n* = 1300) located in the east, most of them were from government sectors, tax bureau, schools, personnel departments, industry and commerce bureaus, bureaus of public security and scientific research institutions. On-the-job civil servants who were 23–60 years of age and had worked for at least over a year were selected as subjects, excluding those who had mental disorders. A total of 5295 completed questionnaires were returned, and 5000 of them were found to be valid, resulting in an effective valid questionnaire response rate of 87.72%. The study protocol has been approved by the Ethics Committee of Xinjiang Medical University (XJMU#2014005). All participants have given written informed consent after receiving details of the study aims and protocol.

### 2.2. Implementation Plans and Quality Control of the Questionnaire

Prior to the survey, we coordinated with the manager of the Department of Human Resources and Social Security of the Xinjiang Uygur Autonomous Region and all of the institutions, and many relevant documents were issued in order to strengthen and improve the co-operation among civil servants. The investigators then carried out the survey along with supporting documents. An announcement was sent to employees explaining that the survey was designed to gain a better understanding of how job strain influences job burnout, mental fatigue and chronic disease. Participants were selected randomly; and participation was voluntary. Respondent interviews were conducted by trained investigators using a structured questionnaire including questions about socio-demographics, job strain, job burnout, mental fatigue and chronic diseases. Once an individual was identified to be suitable and has agreed to participate, he/she was asked to complete the questionnaire independently and anonymously within 20 min during their annual occupational health screening. All investigators were qualified postgraduate students affiliated to the Public Health Department of Xinjiang Medical University and they participated in the preliminary investigation after training offered by the study designer. They described how to fill out the questionnaires, explained the methods and significance of the study, and ensured that the civil servants cooperated positively with a scientific and down-to-earth attitude so that they could complete with integrity every item in the questionnaire. During the survey, all contents were comprehensively checked by the researchers, ensuring that inquiries could be made into anything in doubt about the responses and checked, mistakes corrected, and omissions completed. All investigators met weekly during the data collection period to submit and to discuss any difficulties. All questionnaires were reviewed and encoded by specialized investigators using EpiData version 3.1 (The EpiData Association, Odense, Denmark) to establish a database.

### 2.3. Measures

#### 2.3.1. Demographics

The basic socio-demographic information included sex, ethnicity, educational level, marital status, job title rank, job tenure, region and income level. The types of ethnicity were Han, Uygur, Kazak and other minority. The region consists of two tables: Urban and Non-urban. Other information was grouped respectively into three types: (i) educational level (secondary technical school or below, college, and university or above); (ii) marital status (married, single or divorced/widowed); (iii) job title rank (highest-rank employees; intermediate-rank employees; (iv) lowest-rank employees); (v) job tenure (<10 years, 10–20 years, >20 years); (vi) income level (<3000 yuan per month (<$422), 3000–5000 yuan ($422–$736), >5000 yuan (>$736)).

#### 2.3.2. Assessment of Job Strain

Job strain was assessed based on the Occupational Stress Inventory-Revised (OSI-R) scale, which was divided into three subscales: Occupational Roles Questionnaire (ORQ), Personal Strain Questionnaire (PSQ), and Personal Resources Questionnaire (PRQ). The scale was originally developed by Osipow in 1981 [27] and revised seven times thereafter. Li et al. [28] introduced the scale to China in 1998. In this study, PSQ were used to evaluate job strain within the following categories: vocational, psychological, interpersonal, and physical strain. Subjects responded to each item on a five-point Likert scale ranging from 1 (never) to 5 (always). For PSQ subscales, a higher score indicates a severe level of job strain. We used standard scoring for the PSQ to further classify the job strain group. Raw scores for each dimension were converted to T scores (mean ± SD, 50 ± 10) based on the general population of China [29]. T scores ≥70, between 60 and 69, and ≤59 indicated high, moderate, and low levels of job strain, respectively [30]. The OSI-R has been translated to Chinese and widely used in China, and has been shown to have good reliability and validity in different occupational populations [29,31,32].

#### 2.3.3. Assessment of Job Burnout

Burnout symptoms in this study were assessed by Maslach Burnout Inventory (MBI) questionnaire [18], which includes three parameters: emotional exhaustion, depersonalization, and reduced accomplishment. Emotional exhaustion mainly refers to the weary and worn-out states that result from physical and emotional depletion; it is complicated in particular with emotional symptoms like fatigue, exhaustion, and worries that one’s work will affect his/her own emotional states. Depersonalization mainly reflects a poor social relationship and a negative, indifferent, and evasive attitude towards work, which include manifestations like indifference to the feelings of their subjects, blaming their subjects, and refusing their subjects’ requires. Reduced accomplishment mainly indicates a negative self-assessment of work achievements, such as a sense of incompetence, and a lack of efficiency, morale, and achievement in work.

Each parameter consists of five items, and with a total of 15 items. The questionnaire uses seven magnitudes for scoring each item: 1 representing “completely fitting” and 7 representing “completely unfitting”. The dimension of reduced accomplishment (items 3, 6, 9, 12 and 15) uses reverse scoring. Four levels of job burnout are determined based on the cut-off values (emotional exhaustion score ≥25, personalization score ≥11, and reduced accomplishment score ≥16): no burnout (scored lower than the cut-off values in all three scales), mild burnout (scored no lower than the cut-off value in any one scale), moderate burnout (scored no lower than the cut-off values in any two scales), and severe burnout (scored no lower than the cut-off values in all three scales). The MBI has been translated into Chinese and shows satisfactory reliability and validity [33].

#### 2.3.4. Assessment of Mental Fatigue

Fatigue was assessed with the Multidimensional Fatigue Inventory (MFI-20) [34], which consists of 20 items grouped in five dimensions, including general fatigue, physical fatigue, mental fatigue, reduced activity, and reduced motivation. The current version of the MFI-20 contains 20 statements which cover different aspects of fatigue. The total score of MFI-20 ranges from 20 to 80. The MFI-20 score indicates an individual’s degree of fatigue; a high total score indicates serious fatigue. The study confirmed that the Chinese-version MFI-20 was a reliable and valid instrument for assessing fatigue, and can effectively measure physical and mental fatigue in subjects in China [34].

In the preliminary investigation of this study, 200 office staff members from Xinjiang Medical University were asked to fill out the questionnaires including the Chinese versions of OSI-R, MBI and MFI-20. The reliabilities of the OSI-R, MBI and MFI-20 were assessed by calculating Cronbach’s α, and intraclass correlation coefficient (ICC). The construct validity was analyzed by Root Mean Square Error of approximation (RMSEA), Non-normed fit index (NNFI/TLI), Comparative Fit Index (CFI) and Goodness-of-fit index (GFI) through confirmatory factor analysis. The content validity was measured by item-total Pearson correlations coefficient. The results showed satisfactory reliability and validity (Table 1).

#### 2.3.5. Assessment of Chronic Diseases

According to “Occupational Classification of China” and “Provisional regulations of the state civil servants of China”, civil servants are divided into those in administrative positions, administrative law enforcement, law enforcement, and professional and technical categories, encompassing judges, prosecutors, and police, etc. First, Most civil servants are involved in writing diverse materials, such as briefing information, various notifications, summaries, reports and other official documents. Moreover, they do some daily office work, such as answering telephone calls and forwarding/conveying all kinds of notifications, participating in some kinds of appraisal, and so on. Therefore, civil servants in China are classified as mental workers. Research has showed that hypertension, coronary disease, diabetes, and stroke were diseases with the highest incidence in mental workers [15]. Therefore, the structured questionnaire included four chronic diseases including hypertension, coronary disease, diabetes, and stroke. The participants were asked to answer “yes” or “no” to a question regarding whether they had any chronic disease revealed by diagnosis in hospital or physical examination by a doctor. Thus all answers were self-reported diagnoses.

### 2.4. Statistical Analyses

Data were computerized and analyzed using the Statistical Package Software for the Social Sciences (SPSS version 17, SPSS Inc., Chicago, IL, USA). Descriptive statistics were presented as mean, standard deviation (SD) and range for continuous variables and as number (*n*) and percent (%) for categorical variables. Comparison of the means of two groups was performed using an independent sample *t*-test. ANOVA was used for comparison of the means of multiple groups, which were further examined with the LSD-*t* test for pairwise comparisons, such as comparison of mental fatigue among civil servants with low (a), moderate (b), and high (c) job strain groups (a & b; a & c; b & c). To investigate socio-demographic factor differences, a Chi-square test was used for overall difference among all different job strain level. The Pearson correlation coefficient was used to analyze the strength of correlation between variables.

A multiple linear regression was used to explore explanatory factors for job burnout and mental fatigue which were adjusted for ethnicity. Prior to analyzing the multiple linear regression, the criteria of these methods were reviewed for the nature of the underlying relationships and residuals. Meanwhile, dummy variables were set while some independent variable was categorical with more than two values (there were two unordered categorical variables in our study, the first one was the ethnicity, which included Han, Uygur, Kazak and other minorities, for which we assigned three dummy processing values: Han = 000, Uygur = 001, Kazak = 010, other minority = 100. Another unordered categorical variable was the marital status, which was divided into married, not married or divorced/widowed; we used two dummy processing values: married = 00, not married = 01, divorced/widowed Kazak = 10. We regarded other variables as ordered categorical variables, as they had hierarchical trends, we did not do any dummy processing). Hierarchical regression and binary stepwise logistic regression were used to identify the factors independently associated with chronic disease including sex, ethnicity, educational level, marital status, job title rank, job tenure, region, income level, job strain, job burnout and mental fatigue. All tests were two-sided and conducted at the 5% significance level. *p*-value < 0.05 was considered to be statistically significant.

## 3. Results

### 3.1. Characteristics of Civil Servants

A total of 5000 questionnaires (3049 (61%) from males and 1951 (39%) from females) were deemed valid, for an effective valid questionnaire response rate of 87.72%. The age of the civil servants studied ranged from 23 to 60 years, with an average of 43.4 ± 13.3 years. Approximately half of the sample was of Han ethnicity (2715, 54.3%), university or above in education level (2512, 50.2%) and belonged to the urban group (2528, 50.6%). More than half of the civil servants were married (3145, 62.6%) and lowest-rank employees (3129, 62.6%). The job tenure varied from <10 years (1662, 33.2%), 10~20 years (1981, 39.6%), to >20 years (1357, 27.1%). The income level of 2115 respondents was <3000 yuan/<$422 (42.3%), 1567 earned 3000–5000 yuan/$422–$736 (31.3%), and 1318 declared an income >5000 yuan/>$736 (26.4%).

### 3.2. Correlation between Job Strain and Other Indicators of Civil Servants in Xinjiang, China

When all of the variables were included in the correlation analysis, job strain was found to be positively correlated with job burnout (*r* = 0.625, *p* < 0.05), mental fatigue (*r* = 0.726, *p* < 0.05) and chronic disease (*r* = 0.885, *p* < 0.05), meaning that civil servants under a higher job strain reported higher job burnout, mental fatigue and incidence of chronic diseases. Job burnout also exhibited an obvious positive correlation with mental fatigue (*r* = 0.568, *p* < 0.05), and chronic disease (*r* = 0.325, *p* < 0.05) indicating that civil servants experiencing higher levels of job burnout also had higher levels of mental fatigue and incidence of chronic diseases (Table 2).

### 3.3. Demographics and Factors Associated with Job Strain of Civil Servants

The results demonstrated that there were 334, 1354, and 3312 civil servants in the high, moderate and low-job strain groups, representing a 6.7%, 27.1%, and 66.2% incidence, respectively. It demonstrated that there were 1688 civil servants under high and moderate-job strain, representing a 33.8% cumulative incidence.

Table 3 displays all statistically significant differences in job strain between sex, ethnicity, educational level, job title rank, job tenure, region, marital status, or income level by using chi-square test (*p* < 0.05). The prevalence of high-job strain was higher in females (8.0%) than that in males (5.9%) (*p* < 0.05). It was also the highest in the Uygur ethnic group (9.0%) and the lowest in the Kazak ethnic group (5.0%) (*p* < 0.05). Civil servants who were married (8.9%) had a higher prevalence of high-job strain than their unmarried (2.2%), divorced or widowed (5.3%) (*p* < 0.05) counterparts. The prevalence of high-job strain was the highest in those who have worked for less than ten years in any occupation (9.1%) (*p* < 0.05). Civil servants who were working in urban areas (8.4%) had a higher prevalence of high-job strain than those from non-urban areas (4.9%) (*p* < 0.05). Civil servants whose income level was lower than 3000 yuan were more likely to report high-job strain than those of the high income civil servants (8.0% vs. 5.7% vs. 5.6%). Thus, characteristics including female, Uygur, lower educational level and job title rank, shorter working years, married marital status, and lower income level were associated with a higher prevalence of high-job strain.

### 3.4. Mental Health of Civil Servants with Different Job Strain Levels

#### 3.4.1. Job Burnout Level among Civil Servants

**Figure 1:**
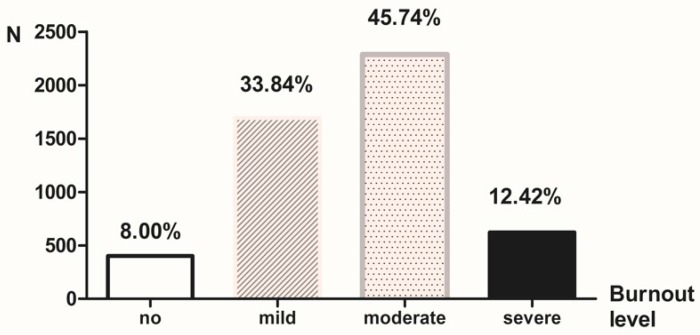
Job burnout level of civil servants in Xinjiang, China.

The results demonstrated that there were 400, 1692, 2287 and 621 civil servants with different job burnout levels in no burnout, mild burnout, moderate burnout and severe burnout groups, representing a 8.00%, 33.84%, 45.74% and 12.42% incidence, respectively (Figure 1).

#### 3.4.2. Comparison of Job Burnout among Civil Servants with Different Job Strain

The prevalence of moderate and severe burnout in civil servants with a high-job strain level were 41.0% and 19.2%, respectively, and the incidence of moderate and severe burnout reached 60.2%. The prevalences of moderate and severe burnout in civil servants with a moderate level of job strain were 47.0% and 9.9%, respectively: the incidence added up to 56.9%. The prevalences of moderate and severe burnout in civil servants with a moderate-job strain level were 45.7% and 12.8%, respectively, amount to 58.5%. There were significant differences in the degree of job burnout among civil servants with different levels of occupational stress (Chi-square value = 32.289, *p* < 0.05), indicating civil servants with a higher level of job strain had higher job burnout level (Table 4).

#### 3.4.3. Comparison of Mental Fatigue among Civil Servants with Different Job Strains

The average score of civil servants’ mental fatigue was 53.7 ± 11.1. We found there were statistically significant differences of mental fatigue scores among groups with low, moderate, and high levels of job strain in civil servants (*F* = 10.426, *p* < 0.05). These were further examined with the LSD-*t* test for pairwise comparisons. LSD-*t* test revealed that mental fatigue scores of civil servants with high level of job strain rose to the peak value which was higher than that of civil servants with a low level of job strain (*t* = 4.653, *p* < 0.05), mental fatigue scores of the group with a moderate level of job strain was also higher than those civil servants with a low level of job strain (*t* = 5.903, *p* < 0.05). Civil servants with a high level of job strain level had higher mental fatigue scores (Table 5).

### 3.5. Chronic Diseases of Civil Servants with Different Levels of Job Strain

#### 3.5.1. Prevalence of Chronic Diseases among Civil Servants

There were 1082 (21.6%) cases of chronic disease in 5000 civil servants. The number of hypertension, coronary disease, diabetes and stroke cases was 552 (11.0%), 373 (7.5%), 101 (2.0%) and 56 (1.1%), respectively. The main chronic diseases civil servants suffered from were hypertension and coronary heart disease, as these two diseases accounted for 18.5% of the total number, accounting for 85.5% of the chronic diseases in civil servants (Table 6).

#### 3.5.2. Comparison of the Prevalence of Chronic Diseases among Civil Servants with Different Job Strains

The results demonstrated that there were 223, 381, and 478 civil servants with chronic disease in the high, moderate, and low-job strain groups, representing a 66.8%, 28.1%, and 14.4% incidence, respectively. The incidence of chronic disease differed significantly (Chi-square value = 607.710, *p* < 0.05; Table 7) according to the degree of civil servants’ job strain, and the higher the degree of job strain was, the higher the incidence of chronic diseases they had.

### 3.6. Risk Factors for Mental Health and Chronic Disease of Civil Servants by Multivariable Regression Analysis

#### 3.6.1. Risk Factors for the Job Burnout and Mental Fatigue by Multivariable Line Regression Analysis

Job burnout and mental fatigue of civil servants were the dependent variables, and individual demographics (e.g., sex, ethnicity, educational level, job title rank, job tenure, region, marital status, or income level), job strain were entered as independent variables (*p* = 0.05) (Table 7).

A model composed of three predictor variables in job burnout which explained 45.0% of its occurrence perceived job strain (β = 0.421, *p* < 0.05) as the variable with the greatest explanatory power. Job strain contributed directly to the occurrence of job burnout. Sex (β = −0.265, *p* < 0.05) and income level (β = −0.162, *p* < 0.05) were inversely associated with job burnout. In other words, in civil servants of female gender, with a lower income level, the higher the level of job strain, the greater was the rate for job burnout (Table 7).

Four factors accounted for 25.0% of the mental fatigue model. Job title rank (β = 0.476, *p* < 0.05) was the variable with the greatest explanatory power. Sex (β = 0.101, *p* < 0.05), rank level (β = −0.198, *p* < 0.05) and job strain (β = 0.213, *p* < 0.05) contributed directly to the occurrence of mental fatigue. Job tenure was inversely associated with mental fatigue, i.e., male civil servants, with a lower job title rank, a higher level of job strain, and a shorter job tenure had a greater rate of mental fatigue (Table 8).

#### 3.6.2. Risk Factors for Chronic Diseases by Binary Logistic Regression Analysis

Chronic disease in civil servants was the dependent variable, and individual demographics (e.g., sex, ethnicity, educational level, job title rank, job tenure, region, marital status, or income level), job strain, job burnout and mental fatigue were entered as independent variables (*p* = 0.05) (Table 8).

Binary logistic regression analysis showed that intermediate-rank employees (OR = 0.442, 95% CI: 0.028–0.634; *p* < 0.05), with job tenure of 10~20 years (OR = 0.632, 95% CI: 0.359–0.989; *p* < 0.05), and low-job strain (OR = 0.657, 95% CI: 0.052–0.698; *p* < 0.05) were associated with significantly lower odds of chronic disease. Risk of chronic disease was higher in civil servants with high-job burnout scores and mental fatigue scores compared with civil servants with lower scores (OR = 1.139, 95% CI: 1.012–3.198; OR = 1.697, 95% CI: 1.097–2.962). In general, civil servants of the lowest rank, having worked for less than ten years, high-job strain, high-job burnout scores and high-mental fatigue scores demonstrated a higher likelihood to suffer from chronic diseases than others (Table 9).

## 4. Discussion

Civil servants in China are under intense work-related pressure. The current study demonstrated that in our sample population there were 1688 civil servants under high and moderate-job strain, representing a 33.8% cumulative incidence. The underlying cause involved the nature of work and the organizational environment that determined the particularity of the work, exposure of civil servants to workplace psychosocial risk factors is different from other occupations, encountering different tension factors in response to specific performance [35]. Specifically, civil servants who are female, of Uygur ethnicity, married, have worked for less than ten years in any occupation, working in an urban area, with an income less than 3000 yuan, reported a higher level of job strain than others. Most of the young civil servants have a short job tenure, a low income, are unmarried, and those who have recently joined the executive authorities are in lower ranks. Civil servants in a low rank who have been employed for a short time [36] are under considerable pressure [37], as there are many factors of instability at their work: the work load is heavy, it is easy to commit errors, and thus wherever they go they are always on the watch. Women’s job strain is significantly higher than that of men, because married women are influenced by the traditional Chinese concept that on the one hand, they should fulfill their duties at the workplace, and on the other hand they should also be dedicated to their families. They spend more time and energy than men, making them deficient in energy and incapable of fully meeting the work requirements. The prevalence of high-job strain was highest in the Uygur ethnic group and lowest in the Kazak ethnic group, probably due to the dissimilar historical backgrounds of different nations. Many Kazak live on traditional grazing lands, and have a more laid-back lifestyle, while the Uygur are a highly motivated and competitive nationality, and so they are more prone to feeling stressed [38].

Under intense work-related pressure, the staff may easily suffer from deteriorated physical and mental health [12], which include disease, fatigue, anxiety, depression, poor ability to work, and even physical and mental failure. This phenomenon is known as job burnout. The great majority of job burnout is attributed to occupational stress, which not only adversely affects the psychological and physical health of workers, but also culminates in low job satisfaction, high absenteeism and poor performance and other organizational behavior issues. We found that civil servants under a high level of job strain had a higher incidence of moderate and severe burnout. The percentage was as high as 60.2%, which is consistent with previous findings on job burnout among civil servants in Southern Brazil [39] and local government employees in Japan [4]. Tense human relationships, heavy work and life pressure and fierce competition contribute to nervous tension in many civil servants. Working alone in an office may indicate a lack of social support, a situation often associated with burnout. With the reform of the civil service system in China, many grass-root civil servants complained that the motivation mechanism of civil servants did not function very well and the mechanism for promotion to higher ranks was considered unfair [40]. Moreover, because the basic assessment of civil servants has become a mere formality and the civil servants rarely enjoy welfare, the incentive pay system does not play a core role. Gradually, job satisfaction of civil servants has fallen to a low level. Civil servants play the most important role in government administration. The work dissatisfaction of grass-roots civil servants directly reduces their work enthusiasm, which causes mental fatigue day after day. The present study uncovered that civil servants with a high level of job strain exhibited higher mental fatigue scores, in line with the findings of Nella [41]. Chronic psychological fatigue, such as reduced interest in work, decreased attention, insomnia, irritability, aggression, and so on, was followed by mental health problems, lowered work efficiency, and more frequent work errors. A correlation between job strain, mental fatigue and chronic disease was found. There is a vicious circle going on involving job strain, burnout and fatigue. To disrupt this vicious cycle, the first and foremost task is to reduce job strain.

In addition, high-intensity work makes civil servants more vulnerable to contract a disease. There were 1082 cases of chronic disease in our participants. Civil servants suffer from chronic diseases which include mainly (85.5%) hypertension and coronary heart disease. Many civil servants are busy and work under stressful conditions. They have sedentary habits and do not play sports. Consequently they are more susceptible to hypertension and hyperglycemia [15]. Our study clearly demonstrates that the degree of civil servants’ job strain differed significantly with the incidence of chronic disease: As the job strain intensified, the higher the incidence of chronic diseases was. In addition, health risk behaviors and habits among civil servants are rampant, including addiction to tobacco and alcohol, lack of exercise and a tendency toward diseases like insomnia [42,43]. Thus, concerns about health, especially predisposition to mental disorders, of special occupational groups, are of tremendous importance and urgency. Marmot [44] reported that the mortality rate of civil servants due to coronary heart disease was above that of the general working population in London. Obesity was found to be more prevalent among Nepalese civil servants than in the general population. The Whitehall II, Helsinki Health, and Japanese Civil Servants studies all suggested that work-related stress, long working hours, and limited time for physical activity of civil servants were associated with chronic diseases and increased mortality rates among civil servants in London, Finland, and Japan, respectively [45]. In the present study, after analyzing risk factors for chronic diseases by multivariable logistic regression, we found that mental fatigue was the main factor that affects the incidence of chronic diseases in civil servants. Fatigue is a persistent and distressing complaint among patients with type 2 diabetes. There is also evidence for a decline in functional capacity due to fatigue in patients with other chronic disorders like cancer and Parkinson’s disease. There are many association with the organizational atmosphere of civil servants, which comprises bureaucracy, politically motivated interference, centralised decision making, paternalism, and disruption of long-term projects due to conflicts of objectives [46]. This atmosphere may place any civil servants at risk of developing health problems. Thus, interventions focused on the individual might not be effective, it is necessary to put an emphasis on preventive strategies which act directly on the system, policy, organization and the ambient atmosphere at the workplace [47,48].

Although the sample size of this study was relatively large, a number of limitations should be considered. First, we collected the information by on-site questionnaire surveys and self-reported mental health and chronic disease were influenced by individual subjective factors, which may have yielded report bias Hence it is necessary to measure some markers of health and conduct psychiatry-based treatments in future research. Second, social factors such as socio-economic characteristics, living environment, policy, organization, social support and atmosphere at the workplace, which may have influenced mental health and chronic disease of civil servants, were not considered in the questionnaire. Third, there are many different types of civil servants in Xinjiang. The situation is different from other regions, therefore, it is not known if these results can be extrapolated to other regions of China or other countries. Fourth, this cross-sectional study could not assess possible causes for these differences, such as the factors leading to higher levels of job strain and poor health in civil servants. In the future, we plan to undertake a longitudinal cohort study to investigate job strain-induced psychological and physiological diseases of civil servants in Xinjiang together with regular health education and health promotion, which could provide more evidence for the importance of reducing the incidence of diseases. This will furnish more data on the significance of understanding strain and health in the civil servants.

## 5. Conclusions

In conclusion, this study revealed that 33.8% of surveyed civil servants were under high and moderate levels of job strain. A higher level of job strain than others was reported by civil servants who were female, Uygur in ethnicity, married, have worked for less than ten years at any occupation, have worked in urban areas, and were earning an income below 3000 yuan ($422). Civil servants under a high job strain had higher burnout and mental fatigue scores. They suffered from chronic diseases which included mainly hypertension and coronary heart disease. The higher the degree of job strain, the higher was the incidence of chronic diseases they had. The effect of job strain on job burnout, mental fatigue and chronic diseases among civil servants was worth noting. Job strain needs interventions with preventive strategies which act directly on the system, policies, organization and the workplace atmosphere.

## Figures and Tables

**Table 1:** Reliability and validity of OSI-R, MBI and MFI-20.

Questionnaire	Reliability		Validity					
			Construct Validity					Content Validity
	Cronbach’s α	ICC	X^2^	RMSEA	TLI	CFI	GFI	Pearson Correlations
OSI-R	0.876	0.851	271.143	0.071	0.862	0.885	0.920	All > 0.6 *
MBI	0.780	0.856	483.011	0.064	0.883	0.903	0.941	All > 0.6 *
MFI-20	0.812	0.843	329.550	0.072	0.880	0.900	0.920	All > 0.6 *

* *p* < 0.05.

**Table 2:** Correlation between job strain and other indicators in civil servants in Xinjiang, China (*r*).

Effect	Job Burnout	Mental Fatigue	Chronic Disease
Job strain	0.625 *	0.726 *	0.885 *
Job burnout	-	0.568 *	0.325 *
Mental fatigue	-	-	0.088

* *p* < 0.05.

**Table 3:** Job strain groups according to different demographic characteristics of civil servants in Xinjiang, China.

Variables		*N*	Job Strain Level			Chi-Square Value	*p*-Value
			High (*n* = 334)	Moderate (*n* = 1354)	Low (*n* = 3312)		
Sex	Male	3049	179 (5.9%)	742 (24.3%)	2128 (69.8%)	44.284	<0.001
	Female	1951	155 (7.9%)	612 (31.4%)	1184 (60.7%)		
Ethnicity	Han	2715	156 (5.8%)	678 (25.0%)	1881 (69.3%)	74.376	<0.001
	Uygur	1506	135 (9%)	407 (27.0%)	964 (64.0%)		
	Kazak	485	24 (5.0%)	199 (41.0%)	262 (54.0%)		
	Other minority	294	19 (6.5%)	70 (23.8%)	205 (69.7%)		
Educational level	Secondary technical school or below	1070	107 (10%)	342 (32.0%)	621 (58.0%)	102.268	<0.001
	College	1418	102 (7.2%)	450 (31.7%)	866 (61.1%)		
	University or above	2512	125 (5%)	562 (22.4%)	1825 (72.7%)		
Job title rank	Grade I	108	5 (4.6%)	17 (15.7%)	86 (79.6%)	180.9	<0.001
	Grade II	1763	48 (2.7%)	165 (9.4%)	1550 (87.9%)		
	Grade III	3129	281 (9.0%)	1172 (37.5%)	1676 (53.6%)		
Job tenure (years)	<10	1662	151 (9.1%)	479 (28.8%)	1032 (62.1%)	74.002	<0.001
	10~20	1981	128 (6.5%)	588 (29.7%)	1265 (63.9%)		
	>20	1357	54 (4%)	287 (21.2%)	1015 (74.8%)		
Region	Urban	2528	213 (8.4%)	632 (25.0%)	1683 (66.6%)	31.581	<0.001
	Non-urban	2472	121 (4.9%)	722 (29.2%)	1629 (65.9%)		
Marital status	Not married	1480	33 (2.2%)	340 (23.0%)	1107 (74.8%)	158.7	<0.001
	Married	3145	281 (8.9%)	844 (26.8%)	2020 (64.2%)		
	Divorced/widowed	375	20 (5.3%)	170 (45.3%)	185 (49.3%)		
Income level	<3000 yuan/<$422	2115	170 (8.0%)	532 (25.2%)	1413 (66.8%)	16.804	0.002
	3000~5000 yuan/$422~$736	1567	90 (5.7%)	432 (27.6%)	1045 (66.7%)		
	>5000 yuan/>$736	1318	74 (5.6%)	390 (29.6%)	854 (64.8%)		

Grade I: highest-rank employees; Grade II: intermediate-rank employees; Grade III: lowest-rank employees.

**Table 4:** Association between job strain and job burnout of civil servants in Xinjiang, China.

Job Strain Level	*N*	Burnout								Chi-Square Value	*p-*Value
		No		Mild		Moderate		Severe			
		*n*	%	*n*	%	*n*	%	*n*	%		
High	334	50	15.0	83	24.9	137	41.0	64	19.2	32.289	<0.001
Moderate	1354	160	11.8	423	31.2	637	47.1	134	9.9		
Low	3312	190	5.7	1186	35.8	1513	45.7	423	12.8		

**Table 5:** Association between job strain and mental fatigue of civil servants in Xinjiang, China.

Job Strain Level	*N*	Mental Fatigue Scores	*F*	*p-*Value
High	334	54.6 ± 12.4 *	10.426	<0.001
Moderate	1354	53.9 ± 10.3 *		
Low	3312	52.5 ± 10.6		

* *p* < 0.05 vs. Low-job strain group.

**Table 6:** Prevalence of chronic diseases in civil servants in Xinjiang, China.

Chronic Disease	Case Number	Morbidity (%)	Proportion of Chronic Disease (%)
Hypertension	552	11.0	51.0
Coronary disease	373	7.5	34.5
Diabetes	101	2.0	9.3
Stroke	56	1.1	5.2
Total	1082	21.6	100

**Table 7:** Association between job strain and mental fatigue in civil servants in Xinjiang, China.

Job Strain Level	*N*	Chronic Disease	
		Case Number	%
High	334	223	66.8
Moderate	1354	381	28.1
Low	3312	478	14.4
Chi-square value		607.710	
*p*-value		<0.001	

**Table 8:** Multiple linear regression analysis of job burnout and mental fatigue in civil servants in Xinjiang, China.

Variables	β	R	*t*	*p*
Job burnout				
Sex	−0.265	0.231	−6.725	<0.001
Income level	−0.162	0.292	−4.913	<0.001
Job strain	0.421	0.601	13.321	<0.001
Mental fatigue				
Sex	0.101	0.125	3.375	0.004
Job title rank	0.476	0.113	14.237	<0.001
Job tenure	−0.198	−0.165	−5.048	<0.001
Job strain	0.213	0.132	4	<0.001

**Table 9:** Binary logistic regression analysis of physiological health of civil servants in Xinjiang, China.

Influence Factor	β	S.E.	Wald	*p-*Value	OR (95% CI)
Rank level					
Grade I	-	-	-	-	1
Grade II	−0.816	0.184	19.657	<0.001	0.442 (0.028, 0.634)
Grade III	0.195	0.240	0.268	0.624	1.216 (0.556, 2.657)
Job tenure (years)					
<10	-	-	-	-	1
10~20	−0.518	0.258	4.044	0.014	0.632 (0.359, 0.989)
>20	0.410	0.592	0.481	0.488	0.664 (0.208, 2.117)
Job strain level					
High	-	-	-	-	1
Moderate	0.128	0.250	0.261	0.61	1.137 (0.696, 1.853)
Low	−0.816	0.245	19.657	0.001	0.657 (0.052, 0.698)
Job burnout scores	0.130	0.026	24.721	<0.001	1.139 (1.012, 3.198)
Mental fatigue scores	0.529	0.335	5.641	0.001	1.697 (1.097, 2.962)

Grade I: highest-rank employees; Grade II: intermediate-rank employees; Grade III: lowest-rank employees; S.E.: Standard error.
